# Cool Runnings – an app-based intervention for reducing hot drink scalds: study protocol for a randomised controlled trial

**DOI:** 10.1186/s13063-016-1521-z

**Published:** 2016-08-03

**Authors:** J. D. Burgess, C. M. Cameron, K. Watt, R. M. Kimble

**Affiliations:** 1 Centre for Children’s Burns and Trauma Research, University of Queensland, Level 7, 62 Graham Street, Brisbane, Queensland 4101 Australia; 2 Wound Management Innovation Cooperative Research Centre, Brisbane, Queensland 4101 Australia; 3 Menzies Health Institute Queensland, Griffith University, Meadowbrook, Queensland 4031 Australia; 4 College of Public Health, Medical and Veterinary Sciences, James Cook University, Townsville, Queensland Australia

**Keywords:** Paediatric, Burns, Scalds, Injury prevention, Intervention, Smartphone applications

## Abstract

**Background:**

Globally, burns are the fifth leading cause of non-fatal children’s injuries, and the leading cause of childhood burns is hot beverage scalds. Although there have been a number of programmes aimed at preventing scalds in children, very few have specifically addressed hot beverage scalds, and fewer have reported a reduction in injury rates. In Australia, hot beverage scalds account for 18 % of all childhood burns – a figure that has remained constant for the past decade.

Innovative new technologies, such as Smartphone applications (apps), present a novel way for delivering individual-level injury prevention messages. The low cost, scalability and broad reach make this technology an ideal channel for health interventions.

One of the latest methods being used in health-related apps aimed at behaviour change is gamification. Gamification uses the gaming principles of rewards, competition and personalisation to engage participants and motivate them towards preferred behaviours.

This intervention will use a Smartphone app-based platform that combines gamification and behaviour-change strategies to increase knowledge and awareness of hot beverage scald risks and burn first aid among mothers of young children.

**Methods/design:**

This is a two-group, parallel, single-blinded randomised control trial (RCT) to evaluate the efficacy of a Smartphone app-based injury prevention intervention. The primary outcome measure is change in knowledge. Change in knowledge is measured in three components: knowledge of correct burns first aid; knowledge of the main cause of burns/scalds in children aged 0–15yrs; knowledge of the main age group at risk for burns/scalds. The secondary outcome measures relate to the gamification methods, measuring participants frequency of engagement with the Cool Runnings app. Queensland-based mothers aged 18+ years who own a Smartphone and have at least one child aged 5–12 months are eligible to participate.

**Discussion:**

To our knowledge, this is the first study to evaluate an app-based delivery of injury prevention messages, and the first study to test the efficacy of gamification techniques in an injury prevention intervention. If this intervention is found to be effective, this RCT will provide a platform for targeting other childhood injury prevention campaigns.

**Trial registration:**

This trial was registered on 14 January 2016 with the Australian New Zealand Clinical Trials Registry (ACTRN12616000019404).

## Background

In Australia, as in most developed countries, hot beverage scalds are the leading cause of burn injuries in young children. Studies from the US, Australia and the UK show hot beverage scalds account for at least 20 % of all childhood burns [1–3]. The high incidence of hot beverage scalds make it an important paediatric public health issue, yet it is often overlooked in research and injury prevention. Although there have been a number of programmes and interventions aimed at the prevention of burns and scalds in children in the past decade, very few have specifically addressed hot beverage scalds, and fewer have reported a reduction in burn and scald injury rates [4, 5]. In Australia, hot beverage scalds account for 18 % of all childhood burns – a figure that has shown no decline in the past 10 years [6]. Given the high incidence of these injuries it is essential that targeted prevention strategies are developed to curb this ongoing paediatric public health issue.

Not only are hot beverage scalds painful, they carry a risk of lifelong psychological stress and physical scarring [7–9]. As well as the physical and emotional consequences, the associated financial costs of managing these injuries on the health care system are also substantial [10]. Therefore, developing targeted prevention strategies to reduce these injuries is essential.

Innovative new technologies, such as Smartphone applications (apps), present a novel way for delivering individual-level injury prevention messages [11], and health behaviour change researchers are harnessing this technology as an intervention tool. The low cost, scalability and broad reach make this technology an ideal channel for health interventions.

The global ownership of Smartphones is growing. In 2014, 81 % of Australian adults owned a Smartphone, and the largest segment of Smartphone users are 18–34 year-olds – the age group being targeted in this intervention [12]. Smartphone ownership goes beyond socioeconomic status boundaries [13]. This medium is personal (individualised and targeted) and portable (always ‘on’), and allows for easy intervention delivery.

One of the latest methods being used in health-related apps aimed at behaviour change is gamification. Gamification is defined as ‘applying game mechanics and game design techniques in a non-game context in order to engage and influence people’s beliefs, attitudes and behaviours’ [14]. Gamification uses the gaming principles of rewards, competition and personalisation to engage participants and motivate them towards preferred behaviours.

This intervention will use a Smartphone app-based platform that combines gamification and behaviour-change strategies to increase knowledge and awareness of hot beverage scald risks and burn first aid among mothers of young children. To our knowledge this is the first study to evaluate a gamified app-based platform targeting injury prevention.

## Methods/design

### Study design

**Fig. 1:**
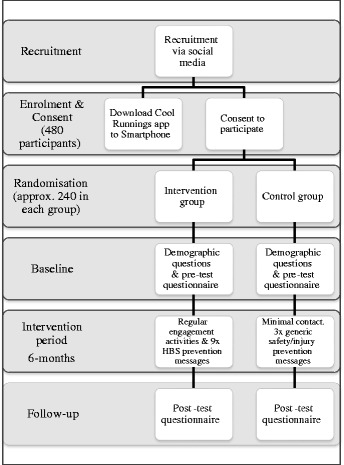
Flow chart of the Cool Runnings trial

A two-group, parallel, single-blinded randomised control trial (RCT) of a technology-based education intervention (Fig. 1).

The protocol for this study has been reported according to the revised Consolidated Standards for Reporting Trials (CONSORT) guidelines [15].

### Study setting

Participants from Queensland, Australia will be recruited via online social media advertisements, specifically through Facebook and Instagram. Treatment (intervention messages) delivery will be Smartphone-based through the Cool Runnings app.

### Ethics approval

This study is registered with the Australian New Zealand Clinical Trials Registry (ACTRN12616000019404) and approved by the University of Queensland Institutional Human Research Ethics Committee (approval number: 2015001652).

### Participants

Inclusion criteria: mothers aged 18+ years of age who own a Smartphone and have at least one child aged between 5 and 12 months at enrolment.

Exclusion criteria: participants will be excluded from this study if they do not meet the inclusion criteria. However, because of the method of recruitment (voluntary response to an online advertisement), it is not possible to be 100 % confident that all participants fulfil the inclusion criteria.

### Selection bias

Selection bias is expected to be minimal because of the fact that 77 % of Australian mothers own a Smartphone [16]. Globally, mothers with children aged under 5 years are the most active on social media [17]. In Australia, 60 % of the Australian population use Facebook and the largest segment are women aged 25–34 years [12].

### Recruitment

Recruitment will be through online advertisements via social media targeted at Queensland- based mothers, aged 18+ years, who own a Smartphone (Android or Apple) and have at least one child aged 5–12 months at the time of recruitment. Facebook advertisements can be specifically targeted only to women who meet the age range, child-age range and geographic location. Potential participants will be given additional information about the study once they click on the link from the online advertisement, and can then download the free app. Participants are shown a participant information page and can consent to the study by clicking on the ‘I have read the study information and I consent to participating in Cool Runnings’. Participants are then randomised to either the control or the intervention group.

### Randomisation

Randomisation will occur through a simple randomisation table created by computer software (i.e. computerised sequence generation). Randomisation will also be stratified by maternal age (18–28 years; 29+ years), based on mean national maternal age [18].

### Blinding

The nature of this study mitigates against full blinding; however, most aspects of this RCT can be blinded. Both participant groups will download the app but are blinded to allocation. The consent form does not mention a control and intervention group (the terms ‘green group’ and ‘blue group’ are used), nor the gamification strategies for each group. Study investigators will assess the outcome data collected from the pre and post questionnaires in a blinded format. Following that point, blinding is not possible for analysing the results of the gamification strategies as they only apply to the intervention group.

All personal and identifiable participant information will be held by the platform licensor, iPug Pty Ltd, and only de-identified information will be given to the study investigators.

### Sample size

A cross-sectional study of knowledge and attitudes toward burn first aid in Queensland by Cuttle et al. [19] showed that 29 % of mothers of children aged 0–4 years in Brisbane correctly identified appropriate burn first aid (cool running water for 20 min). Assuming 90 % power and alpha = .05, in order to detect a 20 % increase in the proportion of mothers who can correctly identify the appropriate burn first aid (type and length) in the intervention group relative to the control group, 240 participants in total are required (120 each in intervention group and control group), with 95 % confidence. This will allow detection of improvement in the intervention group from 29–49 %, with no improvement in the control group.

In order to determine the proportion of participants who correctly identify the main cause of burns/scalds in children under 15 years, and/or the main age group at risk for burns/scalds, a sample size of 96 is required. This will allow detection of the true proportion in this population with 95 % CI and 10 % precision (assuming 50 % prevalence, the most conservative estimate possible). Further, in order to detect a subsequent increase in knowledge of 20 % on both these dimensions for the intervention group relative to the control group, a total sample size of 240 is required (120 in each group). Assuming 50 % loss to follow-up in each group, a total sample of 480 is required (240 intervention; 240 control).

### Intervention

Cool Runnings is an app-based platform that has been developed to implement this intervention. This RCT has an intervention group and an active control group.

#### Intervention group

**Fig. 2:**
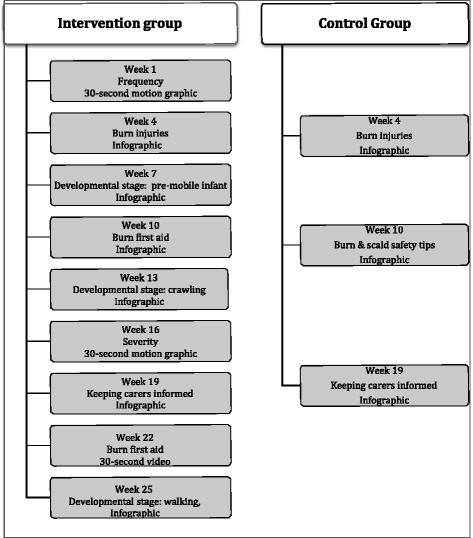
Content calendar for intervention and control group

During the 6-month intervention period the intervention group will receive weekly push notifications from the app inviting them to ‘Play’. Once every 3 weeks, the ‘game’ will feature one of the nine intervention messages; the format of these messages will be either an infographic, motion graphic or a 30-second video (Fig. 2). The intervention messages will focus on hot drink scald risk factors associated with a child’s age and developmental stage, and the correct burn first-aid treatment. During the 2 weeks in-between the intervention messages, participants will be given opportunities to participate in activities that continue to engage them such as posting images of safety devices around the home or answering pop quizzes. The app will record how often participants open, view and engage with the programme. The challenge is to keep participants invested in-between the actual intervention messages. Each time they participate in a ‘game’ they earn points, which means they have a better chance of earning rewards. Participants will accrue points with each full engagement, and once they achieve a certain number of points they will then be eligible to gain a reward. These rewards include shopping and movie vouchers. Participants in this group will also be able to view a ‘leader board’ so they can monitor their points that are being accrued and compare their points with other participating mothers.

#### Control group

This group will access a slightly different app interface, and will only receive three infographic messages over the 6-month intervention period (weeks 4, 10 and 16) (Fig. 2). Two of these messages will be the same as the intervention group receives (about the leading causes of burns in children, and about sharing the infant’s new developmental skills, e.g. standing, climbing, with grandparents and other caregivers). The third infographic is about childhood injuries in the home. There will be no gamification strategies, prizes or incentives offered to the control group; however, they will go in the draw to win one of two iPad Minis for completing both the pre and post questionnaires. It is hoped that the potential to win prizes will minimise loss to attrition. The app will record how often control group participants open, view and engage with the programme. At end of the intervention period the control group will be provided with the same information as the intervention group via their Smartphone app, which will be updated to include the intervention messages. A push message will be sent to participants in the control group inviting them to view the new content.

### Data collection

Both the intervention and control groups will complete demographic questions and two brief questionnaires (pre and post intervention). Demographic information includes: age, area of residence, education, marital status, whether a current smoker, and country of birth. This data will be collected via the Cool Runnings app. Any identifiable information about participants will be stored on a secure, password-protected, encrypted server by the platform licensor (iPug Pty Ltd), and only de-identified information will be passed on to the study investigator. The Licensing Agreement contains a confidentiality clause that states that no information will be released by the licensor to any third parties.

### Primary outcome measure

The primary outcome measure is change in knowledge. Change in knowledge is measured in three components. The first component is knowledge of correct burn first aid. This is measured by an open-ended question (see Table 1). Correct burn first aid knowledge is defined as cool running water for 20 min or more. This is based on clinical evidence of benefit [20, 21]. Any other response reflects incorrect knowledge. The second component is knowledge of the main cause of burns/scalds in children aged 0–15 years (Table 1). This is assessed via response to a multiple-choice question. Any response other than ‘hot drinks’ is coded as incorrect. The third component is knowledge about the main age group at risk for burns/scalds (Table 1). This is also assessed via a multiple-choice question. Any response other than ‘0–2 years’ is coded as incorrect. As such, each of these variables is recoded into dichotomous variable (correct/incorrect). The proportion of correct responses to each of these three knowledge variables will be measured at baseline and post intervention (6 months) using pre and post questionnaires specifically designed for this study [21].

**Table 1:** Outcome measures from pre and post intervention questionnaires for Cool Runnings

Primary outcome questions	Answer options
Hot beverage scald risk:	
What do you think is the main cause of burns and/or scalds in children aged 0–15 years in Australia?	• Bath/taps • Heaters • Hot drinks • Camp fires • Kettle/stovetops • Hair-straighteners • Oven doors • BBQs
What age group do you think is most at risk of receiving this type of burn/scald injury?	• Under 2 years • 2–5 years • 6–10 years • 11–15 years
Burn first-aid treatment:	
What is the recommended first-aid treatment for a burn or scald?	Open-response
When someone has a burn it is recommended that you should apply cold running water. Do you know for how long you should apply cold running water? *NB: this question will be hidden until after the question above is answered*	• 1–5 min • 6–10 min • 11–15 min • 16–19 min • 20 min or more
Secondary outcome measures:	
Frequency of: • App opens • Intervention message views • Pop quiz completions • Photo-sharing	

### Secondary outcome measures

The secondary outcome measures relate to the gamification methods, measuring participants frequency of engagement with the Cool Runnings app (see Table 1).

### Data analysis

All statistical analyses will be conducted using SPSS version 23 (IBM Corporation, Armonk, NY, USA). Descriptive statistics will describe and compare the characteristics of the intervention and control group participants, and pre-knowledge difference. Chi-square analyses will be conducted in order to determine whether there is an increase in the proportion of mothers who can correctly identify the appropriate burn first aid (type and length) (component 1), the main cause of burns/scalds in children under 15 years (component 2), and/or the main age group at risk for burns/scalds (component 3), in the intervention group relative to the control group post intervention.

Increase in knowledge can also be considered a categorical variable (increase versus no increase). Cumulative incidence of increased knowledge in the intervention group versus control group will be calculated, as well as relative risk, absolute risk, absolute risk reduction and numbers-needed-to-treat. This will allow examination of the strength and magnitude of the association between the intervention and change in knowledge.

Where the sample size allows, stratified analyses will be conducted to assess the effect of the intervention in subgroups (age groups; socioeconomic status). In addition, logistic regression will be used to assess increase versus no increase in knowledge of each of the three components of the primary outcome measure. Crude and adjusted analyses will be reported. Methods such as ANCOVA will be used to assess the effect of the intervention while controlling for pre-intervention knowledge, as well a relevant (demographic) confounding factors (this may include, but is not limited to age, socioeconomic status, rurality, education, etc.). Confounding factors will be identified in the descriptive analyses.

## Discussion

By increasing awareness of the frequency and severity of hot beverage scalds, and providing mothers with regular age-relevant messages of the potential risk factors, they will be better equipped to take preventative measures. This intervention also incorporates burn first aid messages. This information is critical because the use of correct burn first aid has such a positive effect on the injury outcome, including faster wound-healing and reduced scarring [22]. It is important that the general public is aware of correct burn first-aid treatment, particularly the parents and carers of young children who have a high incidence of burn injuries.

This study has some limitations. Although the app allows interactivity and some ‘community’ with other mothers sharing and commenting on photo shares in the intervention group, there is no direct contact with participants. Also, to minimise the burden to participants there is a limit on how much information can be collected. Recruitment is via online social media, which limits participants to only those who use this medium. Also, the intervention is only available via a Smartphone app, limiting participants to those who own such a device. However, because of the widespread use of social media and Smartphone ownership in the target group for this trial [12, 16, 17] these limitations are considered minimal. The use of this online approach allows for a broader state-wide reach (or nationally if desired), is cost-effective, and results in faster recruitment compared to traditional methods. In the first 3 weeks alone, 338 participants were recruited to this trial.

The use of gamification in this intervention has the potential to increase engagement and retention of participants, as well as reinforcing the key intervention messages through tactics such as photo-sharing and pop quizzes. Although there are a multitude of health-related apps currently available for chronic disease management, smoking cessation and weight loss [23], to date there has been no research into the efficacy of using gamification in injury prevention interventions.

To our knowledge, this is the first study to evaluate an app-based delivery of injury prevention messages, and the first study to test the efficacy of gamification techniques in an injury prevention intervention. If this intervention is found to be effective, this RCT will provide a platform for targeting other childhood injury prevention campaigns.

## Significance of the study

Unintentional childhood injuries are the leading cause of hospital admissions and emergency department visits among children in the developed world. Therefore, reducing the incidence of childhood injuries is important. One way of achieving this goal could be to harness the popularity and technology of Smartphone apps together with gamification techniques and proven behaviour-change strategies. This study targets a specific, but common childhood injury. If this intervention is found to be effective, this RCT will provide a platform for targeting other childhood injury prevention campaigns, and other public health prevention campaigns generally.

## Trial status

Four hundred and ninety-nine participants have been recruited for this study and the trial period is now active.
